# Combined Effects of Temperature and Seston Concentration on the Physiological Energetics of the Manila Clam *Ruditapes philippinarum*

**DOI:** 10.1371/journal.pone.0152427

**Published:** 2016-03-29

**Authors:** Hee Yoon Kang, Young-Jae Lee, Kwang-Sik Choi, Hyun Je Park, Sung-Gyu Yun, Chang-Keun Kang

**Affiliations:** 1 School of Environmental Science and Engineering, Gwangju Institute of Science and Technology, Gwangju, Republic of Korea; 2 School of Marine Biomedical Science (BK 21 PLUS), Jeju National University, Jeju, Republic of Korea; 3 Department of Marine Bioscience, Gangneung-Wonju National University, Gangneung, Republic of Korea; 4 Department of Science Education, Daegu University, Gyeongsan, Republic of Korea; Institute of Oceanology, Chinese Academy of Sciences, CHINA

## Abstract

The suspension-feeding Manila clam *Ruditapes philippinarum* is a native species of the western Pacific that is now widely distributed around the globe because of its commercial importance. To determine the adaptive physiological responses to changing thermal and nutritional conditions, clearance, filtration, feces production, ammonium excretion, respiration rates, and scope for growth (SFG) were measured in adult clams. The clams were exposed to 24 treatments involving the combination of four water temperatures (8, 13, 18, and 23°C) and six concentrations of suspended particulate matter (SPM: 9.5 to 350.5 mg L^–1^). Physiological rates were standardized by using the mean (480 mg) of tissue dry weights of experimental clams using allometric equations between physiological variables and tissue dry weight. Higher clearance rates were recorded at higher temperatures and lower SPM concentrations, and these rates decreased with increasing SPM concentration at individual temperatures. Consumed energy increased with increasing temperature and SPM concentration, peaking at around 100–200 mg L^–1^ at 18–23°C. Whereas fecal energy was largely determined by SPM concentration, ammonia excretion was mainly governed by temperature. Respiration rate studies revealed a predominant quadratic effect of temperature on the metabolism, indicating a lack of acclimatory adjustment of metabolic rate to rising temperature. SFG values were positive under almost all the treatment conditions and were much higher at higher SPM concentrations (> 45 mg L^–1^), with the highest level being recorded at 18°C and 100–200 mg L^–1^ SPM. Increased filtration rate offset the increased metabolic cost at warm temperatures. Our holistic findings suggest that a high degree of physiological plasticity allows *R*. *philippinarum* to tolerate the wide range of temperatures and SPM concentrations that are found in tidal flats, accounting in part for the successful distribution of this species over a wide variety of geographical areas.

## Introduction

Organisms may respond physiologically to changes in environmental conditions, which in turn can lead to physiological changes in individual organisms, and thus to changes in the properties of natural communities [1]. The ability of organisms to make physiological adjustments in response to ambient environmental changes is crucial for their survival and successful reproduction. Adaptive strategies that are followed by an organism in response to changing environmental conditions can be determined by changes in the rates of individual physiological processes that in turn determine its gain or loss of energy [2]. These adaptive responses can regulate variations in the energetic balances between physiological processes that govern the energy budget, optimizing energy balance between them. Accordingly, information on physiological energetics may provide a clue to understanding the adaptive responses that allow individual organisms to maintain a positive energy balance that is available for both somatic growth and gonadal development.

Temperate tidal flats are exposed to a wide range of seasonal and tidal temperatures, and to large fluctuations in seston concentration (up to 500 mg L^−1^) [3]. A high degree of physiological plasticity in response to such environmental changes may allow tidal flat organisms to survive, grow, and even reproduce in these habitats [4,5]. Infaunal suspension-feeding bivalves are representative members that occur in high densities and thereby serve as an important trophic mediator between lower- and higher-level organisms in tidal flats. It is well known that both their individual physiological functions and integrated energetic balance are associated with both temperature and food availability [2]. Numerous studies have revealed the effects of environmental temperature on total metabolic rate in marine bivalves [6,7,8,9]. When metabolic loss of energy in an animal is elevated under warmer conditions, the animal will increase energy acquisition through food intake to compensate for the loss [10,11,12]. In contrast, animals that lack compensatory adjustment through feeding will encounter a negative energy budget because of elevated metabolic costs [13,14]. However, such an effect of temperature on physiological energetics may be masked by the amount of ingested food due to changes in the availability of food [3,15,16,17]. Accordingly, physiological measurements of the combined effects of temperature and food availability may provide more realistic information on the physiological responses of tidal flat bivalves to a broad range of environmental conditions in their habitats [18,19,20].

The Manila clam, *Ruditapes philippinarum* (Adams & Reeve 1850), is an infaunal suspension-feeding bivalve that inhabits fine sediments in the intertidal and shallow subtidal zones. This clam is native to northwest Pacific coasts, including Korea, Japan, China, and the Philippines, but is now ubiquitous from Asia to Europe and North America, following its introduction for aquaculture and subsequent naturalization [21,22,23]. The high ecological and commercial importance of this species has led to an increasingly large body of research being conducted, which has led to an improved understanding of its growth, physiology, and reproductive biology [24,25,26,27,28,29,30,31,32,33,34,35]. As documented above for other bivalves, previous ecophysiological studies of the Manila clam showed a concurrent elevation of the metabolic rate with increased temperature [33,36]. These authors also observed that the clams offset the elevated metabolic costs by enhancing the rate of ingestion (also filtration) at higher temperatures. Furthermore, several studies have addressed the close relationship between ingestion (assimilation) rates of the Manila clam and concentrations of suspended food in ambient water [17,36,37]. Goulletquer et al. [36] observed a further relationship between the metabolic and filtration rates of the clams. The results of these studies suggest that the physiological rates of the clams can also be regulated by the availability of food, irrespective of temperature. Although information on feeding and the metabolic physiology of tidal flat bivalves is available [3,15,38,39,40,41,42,43], there is a paucity of data on the functional responses of the Manila clam to a broad range of seston concentrations and, in particular, little is known about the combined effects of temperature and food availability on its physiological mechanisms.

The main purpose of the present study was to evaluate the adaptive physiological strategies used by *R*. *philippinarum* to maintain energy balance and maximize energy gain under a wide variety of thermal and nutritional conditions. For this purpose, we examined the energy budget and calculated the scope for growth (SFG) in *R*. *philippinarum* that were exposed to a combination of various temperatures (8, 13, 18, and 23°C) and suspended particulate matter (SPM) concentrations (9.5 to 350.5 mg L^–1^) in ranges typical of native temperate tidal flats, and determined optimal ranges of these variables. We also determined the effect of the combined conditions of temperature and SPM concentration on individual physiological parameters, thereby establishing the SFG.

## Materials and Methods

### Sampling site, harvesting, and acclimation of the clams

The Manila clam *R*. *philippinarum* was collected on a tidal flat of Gomso Bay on the west coast of Korea in January 2012 (35°56′N, 126°54′E). The bay represents one of the most extensive clam farming areas in Korea, and the cultivation area occupies approximately 1450 ha. Water temperature of the coastal area around the bay varies seasonally from 5°C in February to 26°C in August (http://kodc.nfrdi.re.kr). The tide in the bay is semidiurnal, and tidal amplitude averages about 4.3 m, with 5.9 m on spring tides and 2.8 m on neap tides. Strong tidal currents of 115 cm s^–1^ on the flood tide and 150 cm s^–1^ on the ebb tide allow the water within the bay to be well mixed [44]. SPM concentration in the bottom water of the bay displays an extremely broad range from 11.0 to 389.0 mg L^–1^, with a monthly mean range of 109–234 mg L^–1^ [34,45]. Sediment composition of the bay is mostly sand (90%) and silty sand (10%).

Specimens for the experiments were collected during the spring (April). All of the specimens collected for the experiments were transferred to 200 L tanks. The tank was of open-flow design and filled with filtered seawater. Samples were separated into 120 to 150 individuals per tank and progressively acclimated to the appropriate temperature before the experiment. The water temperature of each group was adjusted to 8, 13, 18, or 23°C (Table 1). The temperature in the tanks was increased at a rate of 1°C per day to attain the desired temperature. The four water temperatures were maintained for seven days prior to the experiments. During this acclimation period, the seawater was changed every two days and the experimental clams were daily fed mixed algae with a commercial mixed diet (2 × 106 cells L^–1^, Shellfish Diet 1800®, Reed Mariculture Inc., Campbell, CA) of four marine microalgae (40% *Isochrysis* sp., 15% *Pavlova* sp., 20% *Thalassiosira weissflogii*, and 25% *Tetraselmis* sp.) suitable for shellfish.

**Table 1:** Experimental Conditions.

T (°C)	SL (mm)	DW (mg)	SPM (mg l^-1^)	POM (mg l^-1^)	Energy (J l^-1^)
8	23.88–40.23	167.8–859.6	12.4±1.6	2.8±0.9	11.4±1.8
	26.11–42.02	196.2–1048.0	50.5±3.0	7.3±0.6	33.4±3.4
	23.56–41.18	151.8–867.8	99.5±6.4	15.4±2.3	44.7±6.4
	25.93–43.02	189.3–1021.4	136.5±8.8	25.8±2.9	56.8±3.8
	27.78–43.84	257.0–828.1	199.5±10.1	25.9±2.5	61.2±7.8
	27.72–43.94	207.9–784.7	298.5±14.8	47.3±3.6	85.3±5.9
13	22.59–43.35	153.8–722.4	9.5±1.4	2.4±0.6	7.9±1.3
	26.85–42.25	240.5–1063.7	49.6±2.6	5.2±0.9	29.8±2.1
	28.71–43.54	228.8–877.3	85.5±5.6	10.4±1.1	39.3±3.5
	26.38–39.53	211.6–975.4	150.5±9.2	20.8±1.1	67.4±6.8
	27.57–43.62	207.9–970.3	200.0±11.1	19.0±2.2	70.6±9.5
	22.33–45.21	178.0–827.9	350.5±22.1	64.8±5.0	85.6±12.0
18	25.57–43.84	265.7–606.8	14.6±1.4	3.1±1.2	11.7±1.6
	26.22–49.24	219.4–611.6	58.1±3.7	8.4±0.2	36.1±3.1
	26.17–42.02	199.2–652.5	105.5±5.4	20.3±2.1	54.0±1.4
	16.91–42.20	223.4–662.4	144.8±9.3	27.0±6.7	61.2±6.4
	25.51–46.83	206.1–863.5	204.5±6.7	38.7±4.0	79.1±10.5
	24.85–44.21	189.8–1164.3	324.5±10.7	45.1±7.2	93.2±4.5
23	24.18–40.53	447.8–1139.6	10.1±0.7	1.6±0.3	9.2±3.0
	26.41–42.32	476.2–1328.0	45.7±1.4	7.9±1.6	31.1±1.6
	23.86–41.48	431.8–1147.8	99.6±2.2	14.9±0.6	53.3±2.7
	26.23–43.32	469.3–1301.4	160.4±2.7	19.1±3.5	74.5±3.5
	28.08–44.14	537.0–1108.1	210.7±3.1	25.9±6.4	73.6±7.8
	28.02–44.24	487.9–1064.7	320.6±5.0	40.7±4.5	88.0±4.4

T, water temperature; SL and DW, shell length and dry tissue weight of experimental individuals, respectively; SPM and POM, total quantity and organic fraction of suspended particulate matter supplied to *Ruditapes philippinarum*; Energy, energy value of SPM calculated by energy equivalents (24.0, 17.5 and 39.5 J per 1 mg, respectively) of proteins, carbohydrates and lipids [52].

### Ethics statement

Sample collection and export in the sampling area were permitted by Ministry of Oceans and Fisheries of Korea. Collection and delivery of clam specimens to laboratory was conducted by commercial fishermen, and our field studies did not involve endangered or protected species. Institutional Animal Care and Use Committees approval for this sampling and experimental method was not required; no specific permissions were required for the location and collection because this location is a commercial fishing area.

### Experimental design

To determine the physiological rates of the clams, 300 mL flow-through open cylindrical chambers were used for feeding experiments and 350 mL closed chambers equipped with a magnetic stirrer were used to measure oxygen consumption. For each of the experiments on feeding physiology and respiration, nine experimental chambers each contained a single clam and one was a control chamber without a clam. The chambers were filled with seawater that contained a homogeneous mixture of diet. A mixture of the microalgae mentioned above and surface sediment collected from the bay was used as the experimental diet. For this, thin (< 5 mm) slices of surface sediment were collected and strained through a 100 μm sieve. The clams were starved for 24 h prior to the experiment in filtered seawater and excrement was removed. The SPM concentrations were then controlled at 9.5‒14.6, 49.6‒58.1, 85.5‒105.5, 136.5‒150.5, 200.0‒204.5, and 298.5‒350.5 mg L^–1^ using a peristaltic pump equipped with a 10-channel pump head (BT 100-1L, Baoding Longer Precision Pump Co. Ltd, Baoding, China). The water temperatures were kept constant with water baths at defined experimental temperatures (8, 13, 18, and 23°C). These ranges covered annual variations in water temperature and SPM concentration in the coastal water around the bay. Thus, the set of experiments comprised combinations of four water temperatures and six SPM concentrations. Flow rates in individual chambers were kept constant between 30 and 50 mL min^–1^ depending on animal size. These flow rates were chosen to maintain the percentage of particles in the outflow of the feeding experimental chamber at over 80% of the inflow [46]. Oxygen concentration in each respiration chamber was also kept at over 80% saturation [47]. After acclimation for 6 h under the experimental conditions, all physiological measurements were conducted for 24 h. After evacuation of the gut contents into the filtered water, clam tissues were lyophilized for 72 h and weighed to determine the dry tissue weight (DW).

### Physiological measurements

The clearance rate (CR) was calculated by measuring the difference in particle densities between the outflows of the control and experimental chambers. Particle density was determined every 5 min using a particle counter (PC3400, Chemtrac, Norcross, GA). To calculate the clearance rates of individual clams, the following formula was used: *CR* = {(*C*_1_ − *C*_0_)/*C*_1_ × *F*}, where *C*_1_ and *C*_0_ are particle densities (particles mL^–1^) at the outflow of the control and experimental chamber, respectively, and *F* is the water flow (L h^–1^). The overall mean CR values were calculated for each experimental block.

The filtration (consumption) rate was estimated by collecting particles from the outflows of the control and experimental chambers. To determine the number and nature of the particles filtered by the clams, known volumes of seawater were filtered onto precombusted Whatman GF/F glass fiber filters (47 mm, 0.7 μm pore size) several (4−5) times during the 24-h experiment. The filtration rate (J h^–1^) was then calculated by determining the energy values of the particles that were removed by the clams. Their energy values were obtained from their biochemical composition (i.e., protein, carbohydrate, and lipid content). Protein content was determined using the colorimetric method developed by Lowry et al. [48] after alkaline hydrolysis. Carbohydrates were extracted in 15% trichloroacetic acid and analyzed by the colorimetric method using phenol-sulfuric acid as described by Dubois et al. [49]. Lipid content was analyzed according to the procedure of Marsh and Weinstein [50] after extraction with a mixture of chloroform and methanol [51]. Their energy equivalents were calculated using conversion factors of 24.0, 17.5, and 39.5 J for 1 mg protein, carbohydrate, and lipid, respectively [52]. Pseudofeces production was undetectable during the experiments; if any was present, it was included in the feces.

Feces produced by the clams were collected with a micropipette several (4−5) times over 24 h and placed into a 5 mL precombusted glass tube. The collected fecal materials were rinsed with distilled water, lyophilized, and then weighed. Their biochemical compositions were analyzed using the procedures mentioned above for the filtered particles. The feces production rate (J h^–1^) was estimated by their energy equivalents, calculated using the same conversion factors for protein, carbohydrate, and lipid as described above.

The excretion rate was determined by analyzing ammonia concentrations in the outflows of the control and experimental chambers. Water in the outflow of each chamber was collected several (4−5) times during the 24-h experiment. Ammonia concentration was measured using a standard spectrophotometric method [53]. The excretion rates of ammonia were then calculated from the differences in concentration between the outflows of the control and the experimental chambers. The excretion in individual experimental chambers was converted into energy equivalents (J h^–1^) using a conversion factor of 24.83 J mg^–1^ NH_4_-N [54].

The metabolic rate was obtained by measuring oxygen consumption inside the control and experimental chambers. Oxygen concentrations inside the chambers were measured every second using oxymetric probes (Oxy-10 micro, PreSens-Precision Sensing GmbH, Regensburg, Germany) for 24 h. Details of the procedure used to determine oxygen consumption by continuous monitoring is given elsewhere [55]. The respiration rate was transformed into an energy equivalent (J h^–1^) using a conversion factor of 14.0 J mg^–1^ O_2_ [52].

Scope for growth (SFG) was calculated using the energy balance equation in an individual organism as defined by Winberg [56] and Bayne and Newell [2] as follows: SFG = *A*–(*U* + *R*), where *A* is the absorbed energy, *U* is the energy of ammonia excretion, and *R* is the energy equivalent of metabolic losses. Absorbed energy (*A*) represents *A* = C–*F*, where *C* is the consumed energy and *F* is the energy loss as a result of feces (and pseudoufeces) production. Very few abnormal values for particle density and dissolved concentration probably due to instrumental errors were deleted in the calculation of physiological rates. During the measurement, no individuals stopped their physiological activities. Transiently paused physiological activities were ignored in the rate calculations.

### Statistical analysis

Physiological rates and biometric data (mainly DW) for each experimental block were fitted to the allometric equation:

$$Y=aW^{b},$$

where *Y* is the physiological rate, *W* is DW, and *a* and *b* are the fitted constants. All variances were logarithmically transformed (base 10) and analyzed by least-squares regression analyses between physiological rates and DW values to determine the fitted constants (i.e., *a* and *b*) representing the intercepts and slopes, respectively, of the regression equations. Analysis of covariance (ANCOVA) was employed to test the significance of the differences in slopes of regression equations for individual physiological variables. When there were no significant differences among the estimates of slope (*P* > 0.05), a common slope ($\bar{b}$) and the intercepts were recalculated. The physiological variables were then standardized to the grand mean value of the covariate (0.48 g, a grand mean DW of all the clams in the present experiments) to minimize the confounding effects of variations in body mass from the physiological data [57]. The standardization was performed using the following equation [2]:

$$Y_{s}={\left(W_{s}/W_{e}\right)}^{b}\cdot Y_{e},$$

where *Y*_s_ is the physiological variable of a standard-weight clam, *W*_s_ is its DW, *W*_e_ is DW of the experimental clam, *Y*_e_ is the measured physiological variable of the experimental clam, and *b* is the corresponding allometric coefficient (i.e., $\bar{b}$ in the present experiment).

Lilliefors and Bartlett tests were conducted to determine normality and homoscedasticity of standardized variables, respectively. All the data were square root transformed to meet the assumption of homogeneity of variance. A significant interaction effect of two factors (i.e., water temperature and SPM concentration) in the two-way analysis of variance (ANOVA) meant that group means of the physiological variables were compared among the experimental groups by one-way ANOVA and subsequently by a Tukey *post hoc* test. Stepwise multiple regressions were adopted to assess the effects of the factors and their interactions using a second-order polynomial model as follows:

$$Z=b_{0}+b_{1}T+b_{2}T^{2}+b_{3}S+b_{4}S^{2}+b_{5}T\cdot S,$$

where *Z* represents any of the physiological variables, *T* and *S* are the linear effect, *T*^2^ and *S*^2^ are the quadratic effect, and *T*·*S* is the interaction effect of water temperature and SPM concentration. All of the statistical analyses were performed using a commercially available software package (STATISTICA 12, StatSoft Inc., Tulsa, OK).

## Results

Allometric relationships between individual physiological rates and DW of *R*. *philippinarum* were found to be significantly positive at all the experimental temperatures and SPM concentrations (Table 2). ANCOVA tests for physiological rates (i.e., clearance, consumption, excretion, feces production, and respiration) revealed no significant differences among the slopes of almost all the regressions for individual physiological parameters (*F*_23, 167_ = 1.563, *P* = 0.058; *F*_22, 161_ = 1.532, *P* = 0.070; *F*_23, 167_ = 1.502, *P* = 0.076; *F*_23, 168_ = 1.078, *P* = 0.375; *F*_23, 167_ = 0.819, *P* = 0.705, respectively). Common slopes ($\bar{b}$) of regressions for these individual parameters were then estimated to be 0.792 (± 0.023, SE), 0.412 (± 0.021), 0.428 (± 0.016), 0.624 (± 0.025), and 0.506 (± 0.026), respectively, providing weight exponents to standardize the physiological rates to a given mass (a standard clam of 0.48 g DW) in the present study.

**Table 2:** Regression coefficients of physiological rate (Y, J) and tissue dry weight (X, g) for *Ruditapes philippinarum* following allometric equation Y = *a*X^*b*^.

Physiological factor	Fs	df	Significance (*P*)	$\bar{b}$ (SE)
Clearance	1.563	22, 167	0.058	0.792 (0.023)
Filtration	1.532	22, 161	0.070	0.412 (0.021)
Ammonia excretion	1.502	23, 167	0.076	0.428 (0.016)
Feces production	1.078	23, 168	0.375	0.624 (0.025)
Respiration	0.819	23, 167	0.705	0.506 (0.026)

Y = l h^−1^ clearance rate, J h^−1^ filtration, feces production, oxygen consumption, and 10^−3^ J h^−1^ ammonia excretion. $\bar{b}$ represents common slopes of individual physiological factors obtained from analysis of covariance (ANCOVA) to test significance of differences in slope between 24 experimental blocks (4 temperatures × 6 suspended particulate matter concentrations). n = 9 for each experiment. An experimental-block dataset for filtration measurement was excluded from ANCOVA test because the regression slope was different from others.

**Fig 1:**
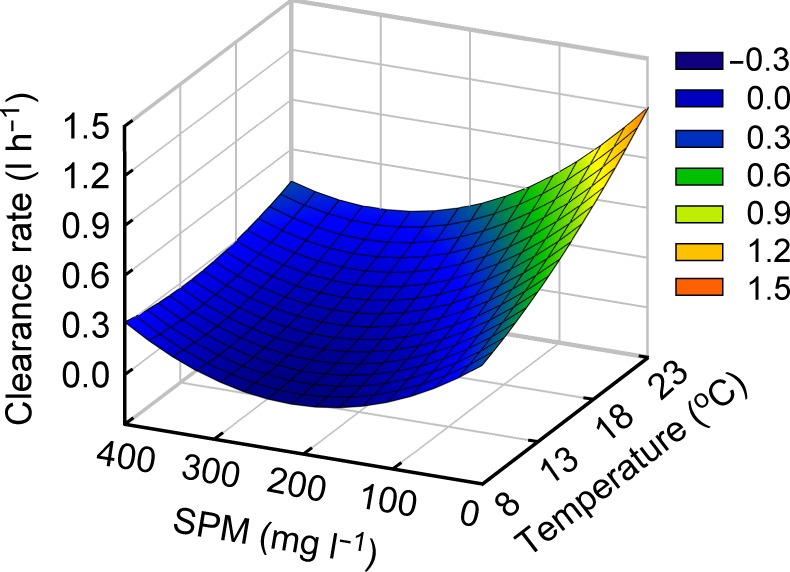
Surface plot of response of mean clearance rate of the Manila clam. Mean clearance rate (CR) represents values of a 0.48 g weight-standardized Manila clam *Ruditapes philippinarum* in experimental combinations of water temperatures (T) and suspended particulate matter (SPM) concentrations (S) based on the following mathematical model: CR = 0.2722 + (0.0052 T)–(0.0030 S) + (0.0016 T^2^) + (9.3629E^-6^ S^2^)–(0.0001 T S), R^2^ = 0.943 (*P* < 0.0001).

Mass specific rates of all physiological parameters of a standard clam differed significantly between treatments with different combinations of temperature and SPM concentration (Table 3). The responses of individual physiological parameters to such different combinations displayed different patterns. A gradual reduction in the clearance rate (CR) with increasing SPM concentration was detected at individual temperatures. In contrast, there was a general rise in the CR with increasing temperature at analogous SPM concentration levels. The CR increased by more than 50% for each 5°C increase in temperature. The multiple regression analysis revealed a significant quadratic effect (*P* = 0.0003) of temperature and the interacting effect (*P* < 0.0001) of both environmental factors (Table 4; Fig 1). These effects explained 45.5% and 38.6%, respectively, of the variation.

**Table 3:** Mean (± SD) values of physiological rates of a weight-standardized (0.48 g dry tissue weight) Manila clam *Ruditapes philippinarum*.

T (°C)	SPM (mg l^−1^)	CR (l h^−1^)	C (J h^−1^)	F (J h^−1^)	U (mJ h^−1^)	R (J h^−1^)	SFG (J h^−1^)
8	12.4	0.27 ± 0.02 ^gh^	2.71 ± 0.48 ^a^	0.06 ± 0.01 ^a^	0.17 ± 0.01 ^a^	1.6 ± 0.3 ^a^	1.1 ± 0.6 ^a^
	50.5	0.22 ± 0.04 ^fg^	5.18 ± 0.70 ^cde^	0.09 ± 0.01 ^ab^	0.21 ± 0.01 ^b^	1.8 ± 0.4 ^ab^	3.3 ± 1.1 ^cd^
	99.5	0.11 ± 0.02 ^c^	5.11 ± 0.73 ^cd^	0.14 ± 0.02 ^cdefg^	0.33 ± 0.02 ^de^	2.5 ± 0.5 ^bcde^	2.5 ± 0.8 ^bc^
	136.5	0.09 ± 0.01 ^bc^	6.50 ± 0.77 ^efg^	0.16 ± 0.03 ^efghi^	0.32 ± 0.03 ^d^	2.2 ± 0.3 ^abcd^	4.1 ± 0.7 ^def^
	199.5	0.06 ± 0.01 ^b^	6.09 ± 0.33 ^def^	0.19 ± 0.02 ^ghij^	0.27 ± 0.05 ^c^	2.5 ± 0.3 ^bcdef^	3.4 ± 0.3 ^cd^
	298.5	0.03 ± 0.00 ^a^	5.84 ± 0.46 ^def^	0.24 ± 0.02 ^jk^	0.34 ± 0.04 ^def^	3.6 ± 0.4 ^ij^	2.0 ± 0.4 ^ab^
13	9.5	0.63 ± 0.06 ^k^	4.19 ± 0.74 ^bc^	0.10 ± 0.02 ^abcde^	0.39 ± 0.05^f^	2.1 ± 0.3 ^abc^	2.0 ± 0.7 ^ab^
	49.6	0.45 ± 0.04 ^j^	7.72 ± 1.10 ^ghi^	0.13 ± 0.02 ^bcdef^	0.39 ± 0.04^f^	2.4 ± 0.3 ^bcde^	5.2 ± 1.2 ^fgh^
	85.5	0.22 ± 0.02 ^ef^	8.23 ± 1.35 ^hi^	0.14 ± 0.02 ^bcdef^	0.38 ± 0.03^ef^	2.8 ± 0.3 ^cdefgh^	5.3 ± 1.4 ^fgh^
	150.5	0.17 ± 0.02 ^d^	7.77 ± 1.22 ^ghi^	0.16 ± 0.02 ^aefghi^	0.51 ± 0.05^g^	2.7 ± 0.4 ^cdefg^	5.0 ± 1.1 ^efg^
	200	0.09 ± 0.02 ^bc^	8.66 ± 0.94 ^i^	0.16 ± 0.02 ^efgh^	0.46 ± 0.02 ^g^	3.0 ± 0.6 ^defghi^	5.5 ± 1.1 ^fgh^
	350.5	0.07 ± 0.01 ^b^	7.04 ± 0.71 ^fgh^	0.21 ± 0.03 ^hij^	0.64 ± 0.04 ^h^	3.4 ± 0.6 ^ghij^	3.5 ± 0.9 ^cde^
18	14.6	0.86 ± 0.06 ^l^	5.88 ± 0.61 ^def^	0.11 ± 0.02 ^abcdef^	0.46 ± 0.03 ^g^	3.1 ± 0.4 ^efghi^	2.7 ± 0.5 ^bc^
	58.1	0.47 ± 0.05 ^j^	14.23 ± 1.68 ^kl^	0.10 ± 0.01 ^abcd^	0.47 ± 0.04 ^g^	3.3 ± 0.5 ^fghij^	10.9 ± 1.5 ^k^
	105.5	0.37 ± 0.04 ^i^	19.66 ± 1.32 ^op^	0.16 ± 0.03 ^efgh^	0.61 ± 0.05 ^h^	2.9 ± 0.4 ^cdefghi^	16.7 ± 1.3 ^m^
	144.8	0.33 ± 0.06 ^hi^	17.82 ± 1.45 ^no^	0.14 ± 0.03 ^cdefg^	0.64 ± 0.04 ^h^	4.0 ± 0.7 ^j^	13.7 ± 1.4 ^l^
	204.5	0.27 ± 0.03 ^gh^	20.93 ± 1.79 ^p^	0.27 ± 0.03 ^k^	0.93 ± 0.03 ^j^	3.3 ± 0.5 ^fghij^	17.9 ± 1.0 ^m^
	324.5	0.18 ± 0.03 ^de^	13.06 ± 1.41^k^	0.36 ± 0.03 ^l^	0.74 ± 0.04 ^i^	3.6 ± 0.5 ^hij^	9.2 ± 1.5 ^jk^
23	10.1	1.33 ± 0.06 ^m^	3.86 ± 0.50 ^b^	0.08 ± 0.01 ^ab^	0.58 ± 0.05 ^h^	5.3 ± 0.5 ^k^	−1.6 ± 0.7
	45.7	0.86 ± 0.05 ^l^	10.87 ± 1.34 ^j^	0.09 ± 0.01 ^abc^	0.59 ± 0.03 ^h^	5.2 ± 0.7 ^k^	5.6 ± 1.1 ^fgh^
	99.6	0.63 ± 0.05 ^k^	15.36 ± 1.05 ^lm^	0.11 ± 0.01 ^abcde^	0.73 ± 0.03 ^i^	8.4 ± 0.7 ^lm^	6.8 ± 1.2 ^hi^
	160.4	0.48 ± 0.03 ^j^	19.30 ± 1.26 ^op^	0.15 ± 0.02 ^defg^	0.72 ± 0.05 ^i^	8.2 ± 0.6 ^l^	11.0 ± 1.4 ^k^
	210.7	0.46 ± 0.04 ^j^	17.28 ± 1.03 ^mno^	0.17 ± 0.02 ^fghi^	0.77 ± 0.04 ^i^	9.3 ± 0.6 ^n^	7.8 ± 1.2 ^ij^
	320.6	0.38 ± 0.04 ^i^	15.80 ± 0.67^lmn^	0.21 ± 0.02 ^ij^	0.77 ± 0.05 ^i^	9.2 ± 0.4 ^mn^	6.4 ± 0.6 ^ghi^

CR, clearance rate; C, consumed energy; U, excretion energy; F, feces energy; R, respiration energy; and SFG, scope for growth. 0.48 g dry tissue weight is a grand mean of all experimental clams. The Manila clams were maintained in combinations of four water temperatures (T) and six suspended particulate matter (SPM) concentrations. The same superscript letters within the same column represent no significant differences (P < 0.05).

**Table 4:** Determination coefficient (R^2^) of multiple regressions for physiological rates of a standard-weight (0.48 g dry tissue weight) Manila clam *Ruditapes philippinarum* in 24 combinations of water temperatures (T) and suspended particulate matter (SPM) concentrations (S).

	**CR**		**C**		**F**	
	**R²**	***P***	**R²**	***P***	**R²**	***P***
T	0.00018	0.81677	0.44472	0.00037	0.03687	0.10565
S	0.05317	0.00049	0.08361	0.01419	0.70466	0.00000
T²	0.45528	0.00030	0.03418	0.08389	0.00172	0.72941
S²	0.04836	0.00775	0.13689	0.00687	0.00071	0.82564
T·S	0.38592	0.00000	0.11687	0.02767	0.00116	0.77252
Total	0.94290	0.00000	0.81626	0.00000	0.74511	0.00000
	**U**		**R**		**SFG**	
	**R²**	***P***	**R²**	***P***	**R²**	***P***
T	0.67379	0.00000	0.09315	0.00471	0.16589	0.04822
S	0.17397	0.00008	0.00355	0.42950	0.10200	0.03051
T²	0.02808	0.04608	0.71105	0.00000	0.15586	0.03940
S²	0.01518	0.12019	0.00419	0.38666	0.18127	0.01109
T·S	0.00220	0.54997	0.09015	0.00052	0.06193	0.17168
Total	0.89323	0.00000	0.90209	0.00000	0.66696	0.00073

CR, clearance rate; C, consumed energy; U, excretion energy; F, feces energy; R, respiration energy; and SFG, scope for growth. 0.48 g dry tissue weight is a grand mean of all experimental clams. T and T^2^, lineal and quadratic effect of water temperature; S and S^2^, lineal and quadratic effect of SPM concentration; T·S, interaction effect of water temperature and SPM concentration.

**Fig 2:**
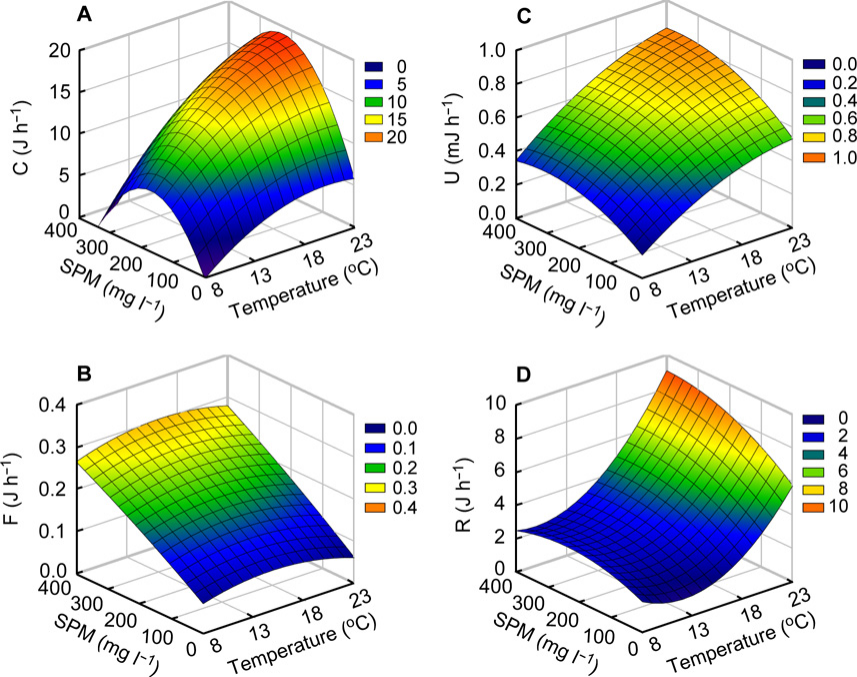
Surface plots of responses of mean physiological rates of the Manila clam. (A) Mean consumed energy, (B) Feces energy, (C) excretion energy energy, and (D) Respiration energy. Mean consumed energy (C), feces energy (F), excretion energy energy (U), and respiration energy (R) represent values of a 0.48 g weight-standardized Manila clam *Ruditapes philippinarum* in experimental combinations of water temperatures (T) and suspended particulate matter (SPM) concentrations (S) based on the following mathematical model: C = –11.1200 + (1.6938 T) + (0.0723 S)–(0.0416 T^2^)–(0.0003 S^2^)+(0.0019 T S), R^2^ = 0.816 (*P* < 0.0001); F = –0.0182 + (0.0154 T) + (0.0005 S)–(0.0005 T^2^)–(1.6779E–7 S^2^) + (3.9736E–6 T S), R^2^ = 0.745 (*P* < 0.0001); U = –0.3274 + (0.0692 T) + (0.0013 S)–(0.0014 T^2^)–(2.3435E–6 S^2^) + (1.6394E–5 T S), R^2^ = 0.893 (*P* < 0.0001); R = 6.8283 –(0.9390 T) + (0.0060 S) + (0.0388 T^2^)–(1.9534E–5 S^2^) + (0.0004 T S), R^2^ = 0.902 (*P* < 0.0001).

Consumed (filtration) energy increased with increasing temperature and SPM concentration, but showed a slight reduction when both factors increased from 18 to 23°C and from 199.5–210.7 to 298.5–350.5 mg L^–1^, respectively (Table 3; Fig 2A). Accordingly, consumed energy values were the lowest (2.71–3.86 J h^–1^) at the lowest SPM concentrations (10.1–12.4 mg L^–1^) at 8 and 23°C, and peaked (17.28–20.93 J h^–1^) at 105.5 to 210.7 mg L^–1^ at both 18 and 23°C. The regression analysis showed a direct effect of temperature (*P* = 0.0004) as well as a significant quadratic effect of SPM concentration (*P* = 0.007) on consumed energy (Table 4). The interaction between both factors on consumed energy was weak but significant (*P* = 0.028). The sum of these effects explained 69.8% of the variation. Feces energy was significantly affected by SPM concentration (Table 3; Fig 2B). Its values were very low (0.06–0.11 J h^–1^) at the lowest SPM concentrations and increased to 0.21–0.36 J h^–1^ at the highest SPM concentrations at all four temperatures. The regression analysis revealed a direct and significant effect of SPM concentration (*P* < 0.0001) on feces energy (Table 4). This effect explained 70.5% of the variation. Excretion and respiration energy showed a general pattern: they increased with increasing temperature and SPM concentration (Table 3; Fig 2C and 2D), with the lowest values (0.17–0.34 mJ h^–1^ and 1.6–3.6 J h^–1^, respectively) at 8°C and the highest (0.58–0.77 mJ h^–1^ and 5.3–9.3 J h^–1^, respectively) at 23°C. These parameters showed slightly different response patterns. Excretion energy was directly related to temperature (*P* < 0.0001) and SPM concentration (*P* < 0.0001), the sum of which explained 84.8% of the variation (Fig 2C; Table 4). In contrast, respiration energy was characterized by a high significance in the quadratic effect (*P* < 0.0001) of temperature that explained 71.1% of the variation (Fig 2D; Table 4).

**Fig 3:**
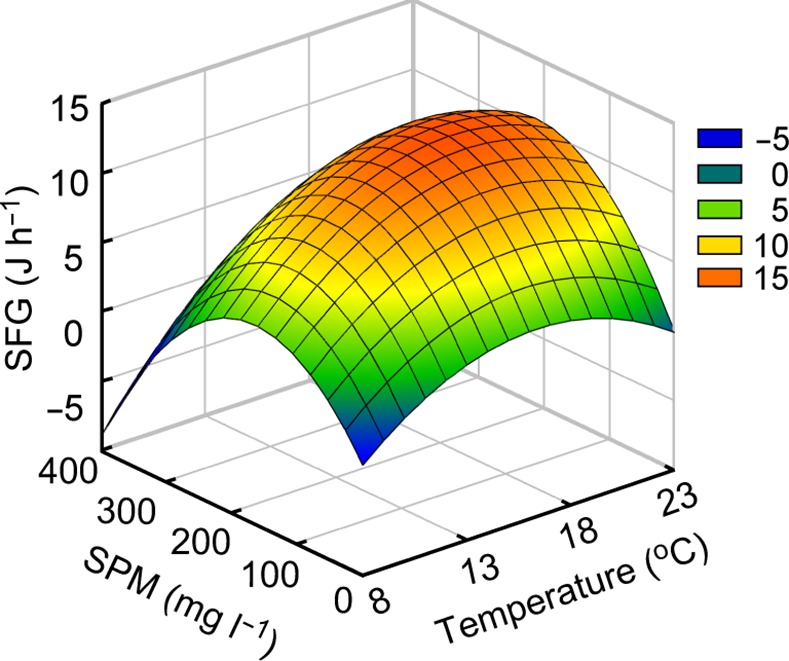
Surface plot of response of mean scope for growth of the Manila clam. Mean scope for growth (SFG) represents values of a 0.48 g weight-standardized Manila clam *Ruditapes philippinarum* in experimental combinations of water temperatures (T) and suspended particulate matter (SPM) concentrations (S) based on the following mathematical model: SFG = –18.1652 + (2.6470 T) + (0.0664 S)–(0.0809 T^2^)–(0.0002 S^2^) + (0.0015 T S), R^2^ = 0.667 (*P* = 0.0007).

Scope for growth (SFG) was found to be negative (–1.6 J h^–1^) at the lowest SPM concentration of 10.1 mg L^–1^ at 23°C but positive for all the other treatments, displaying maximum levels of 13.7–17.9 J h^–1^ at 105.5–204.5 mg L^–1^ at 18°C (Table 3; Fig 3). The regression analysis showed that temperature and SPM concentration had a significant effect (*P* = 0.048 and 0.031, respectively) on the SFG and so did quadratic effects of both factors (*P* = 0.039 and 0.011, respectively), with the sum of these effects explaining 60.5% of the variation (Table 4).

## Discussion

The physiological rates (i.e., clearance, filtration, feces production, excretion, and respiration) of *R*. *philippinarum* were positively related to body weight at all the experimental temperatures and SPM concentrations, indicating generally higher rates in the larger individuals. Individual weight exponents ($\bar{b}$) for physiological rates in allometric equations were identical for almost all the experimental combinations of temperature and SPM concentration (Table 1). This result indicates that small and large individuals are invariably subjected to the same levels of physiological reactions (i.e., changes in rates) under the different experimental combinations. Therefore, comparisons of the physiological rates and, thereby, the SFG of clams of a particular weight between combinations of temperature and SPM concentration enable us to elucidate the combined effect of the two parameters on the energetics. On the other hand, our estimations of weight exponents corresponded to the lower limit of ranges for suspension feeders [2,58,59]. The relatively low exponent value for the filtration rate in the present study is likely to reflect more pronounced reduction of feeding in larger individuals than in smaller individuals in the experiments with natural particulates compared with cultured algal diets, as previously reviewed [2, 58]. This also points out the important effect of seston organic content on bivalve feeding [59]. Furthermore, the lower exponent value for the filtration rate (0.412) than for the respiration rate (0.504) is consistent with the results obtained with other suspension feeders [2].

### Energy acquisition processes: clearance rate (CR) and filtration rate

The CR and consumed energy (i.e., filtration rate) of *R*. *philippinarum* demonstrated that these species can maintain suspension feeding over a broad range of temperatures between 8 and 23°C, and SPM concentrations between 9.5 and 350.5 mg L^–1^. The temperature and SPM concentration had a direct and interacting effect on the clearance and filtration rates (Table 3). The results of the present study suggest that *R*. *philippinarum* can physiologically regulate feeding and thereby energy acquisition under a broad range of environmental conditions, as exemplified by other intertidal bivalves [3,5,15]. A rise in the CR with increasing temperature and its gradual reduction with increasing SPM concentration was observed in other feeding experiments for *R*. *philippinarum* [30,33,60]. The present study evaluated the combined effect of the two parameters on the clam CR. The CR of a 0.48 g (in DW) standard clam ranged from 0.03 to 1.33 L h^–1^ (0.06–2.38 L g^–1^ h^–1^), peaking at 23°C and 10.1 mg L^–1^, followed by the second highest values of 0.86 L h^–1^ (1.54 L g^–1^ h^–1^) at 23°C and 45.7 mg L^–1^ (also at 18°C and 14.6 mg L^–1^). The high CR values at 18 and 23°C fell well within the range of values (0.6–10.3 L g^–1^ h^–1^) reported for other temperate bivalves [61] but below the range at 8 and 15°C (except 9.5 and 49.6 mg L^–1^). The clam CR values in the present study were lower than those previously measured for *R*. *philippinarum* (2.5 L g^–1^ h^–1^ [30]; 0.9–5.1 L g^–1^ h^–1^ [33]), probably reflecting experiments performed under different dietary conditions (composition of mixed semi-natural *vs*. fresh algal diets and particle concentrations). Low CRs of 0.04–0.10 L g^–1^ h^–1^ (*Crassostrea corteziensis*) and 0.33–0.56 L g^–1^ h^–1^ (*Anaara tuberculosa*) were also observed for other bivalve mollusks depending on the experimental conditions [62]. Considering the previous finding that the CR of *R*. *philippinarum* increases in the temperature range of 12−21°C and decreases in the range of 21−30°C [30], the present result supports the conclusion that this species may have an optimal temperature range of between 18 and 23°C for CR (cf. 20−25°C [33]; 12−20°C [36]). However, despite a marked decline at low temperatures and high SPM concentrations, there was no complete cessation of CR in the ranges investigated in our experiments, even at 5°C [33] and 25°C [36].

Although a clear inflexion point was not observed for the CR within our experimental ranges of the two parameters, the highest levels (17.28–20.98 J h^–1^) of consumed energy (i.e., filtration rate) in a standard clam were found at around 100–200 mg L^–1^ at both 18 and 23°C. These results suggest that the filtration rate of the clam may not necessarily reflect its CR [43,63]. Filtration of marine invertebrates increases concurrently with temperature up to an optimal level and then decreases significantly beyond this level [6,14,36,47]. A similar trend in relation to increasing SPM concentration has also been demonstrated for intertidal and estuarine bivalves [2,3,15,30,40]. In the present study, a reduction in the rates of clam filtration appears to be more pronounced when tested at high SPM concentrations than at high temperatures, as indicated by a lineal effect of temperature and a significant quadratic effect of SPM concentration on consumed energy. Indeed, the maximum rates of filtration by the clam were observed within the SPM range (100–200 mg L^–1^) found in its natural intertidal habitats [34]. So far, the upper or lower tolerance limits of the two parameters, at which a cessation of feeding of the clam occurs, are unclear. The present findings would suggest that *R*. *philippinarum* persists in suspension feeding over a broad range of temperatures and SPM concentrations, and can regulate feeding activity to maximize energy acquisition in the relatively high SPM conditions of the intertidal area.

### Energy expenditure processes: feces production, excretion, and respiration

Fecal energy of the Manila clam measured in this study was independent of temperature and, instead, was largely determined by SPM concentration. However, the energy fraction (less than 5% of filtered energy in the present study) rejected by fecal materials of the clam was much lower than those reported for marine invertebrates [2,14,33]. This probably arose from the low levels of organic content (ca. 16%) in the SPM used in the present feeding experiments (Table 1). On the other hand, although the energy loss through ammonia excretion was mainly determined by temperature, its absolute values accounted for a negligible proportion (< 0.02%) of the energy absorbed by the clam. A close relationship between temperature and rate of ammonia excretion is a general phenomenon in *R*. *philippinarum* [33]. Therefore, feces production and ammonia excretion by the clam showed distinct patterns of responses to the combinations of temperature and SPM concentration. These patterns also differed according to the feeding strategies (i.e., CR and filtration) of the clam. Although SPM concentration- and temperature-dependent trends in feces production and ammonia excretion, respectively, were observed in the present experiments, these responses would not lead to distinct patterns of absorbed and assimilated ratios (data not shown) from that of filtration because of the low contribution of these rejection/excretion processes to energy expenditure of the clam, as mentioned above.

The response of respiration rate to the combinations of temperature and SPM concentration revealed a predominant quadratic effect of temperature on the metabolic energy expenditure of *R*. *philippinarum*, suggesting an increase in the metabolic cost of maintenance with increasing temperature, as generally observed in many bivalve mollusks [2,6,9]. In addition, considering the lineal effect of temperature on the rate of filtration, the higher respiration energy at high temperature would also include the increased metabolic costs of feeding (i.e., digestion, absorption, and growth) [42,64]. Indeed, *R*. *philippinarum* displayed a broad range of respiration energy (2.2–13.4 J g^–1^ h^–1^) corresponding to the range of values (1.1–18.6 J g^–1^ h^–1^) reported for temperate bivalves [61]. This kind of response to a broad range of environmental conditions differs from the metabolic adjustment that allows some other bivalves to maintain their respiration energy within a narrow range (*Ostrea edulis* [8]; *Pinctada mazatlanica* [9]; *Anadara tuberculosa* [65]). As a result, the Manila clam may need further compensatory adjustment to optimize their energy balance because of the lack of acclimatory adjustment of metabolic rate to rising temperature [10,11].

### Scope for growth (SFG)

The physiological responses of the Manila clam *R*. *philippinarum* presented herein highlight their highly plastic physiological processes with respect to energy acquisition and expenditure. These animals have developed critical adaptive strategies to colonize, survive, and grow in intertidal areas that are characterized by large fluctuations in environmental conditions. The SFG integrates the overall physiological responses of clams to combinations of temperature and SPM concentration, displaying a range of –1.6 to 17.9 J^–1^ h^–1^ in a standard individual. These estimations correspond to 1.3–23.1 J g^–1^ h^–1^ except for only one negative value (–2.6 J g^–1^ h^–1^), falling well within the range reported for temperate bivalve mollusks [61]. The highest SFG (> 13 J^–1^ h^–1^) was recorded in clams that were held at 18°C and around 100–200 mg L^–1^, meeting the highest filtration rate (20.93 J^–1^ h^–1^) and relatively low metabolic rate (3.3 J^–1^ h^–1^) compared with clams held at 23°C. This combination provided the best conditions for growth of the clam, and they are consistent with temperatures that are typical of its fast-growing spring period and the SPM range in its natural intertidal habitats [34]. Negative SFG was recorded for clams held in an environment of 23°C and 10.1 mg L^–1^. Except for the only case of negative SFG, the clams exhibited positive SFG across all experimental combinations of temperature and SPM concentration, indicating that they can adapt successfully to a broad range of environmental conditions on the intertidal flats that they inhabit.

Although their SFG was optimal at 18°C and 100–200 mg L^–1^, the clams showed different patterns of responses in their physiological parameters to combinations of temperature and SPM concentration. Indeed, the positive energy balance of the clam reflected the reduction in metabolic costs at lower temperatures (8–13°C). In contrast, marked elevations in consumed energy (filtration rate) and rapidly increased metabolic costs at higher temperatures (18–23°C) resulted in higher SFG than at lower temperatures. This explanation is consistent with the increased SFG at higher SPM concentrations (> 45 mg L^–1^). The SFG increase resulting from such responses to rising temperature was undetectable at the lowest SPM concentrations of individual temperatures and even fell to a negative value at 23°C. These results indicate that the energy consumed by the clam increases to a much higher degree than is compensated for by increased metabolic costs under higher temperature and SPM concentration conditions, but not at the lowest SPM concentration. In addition, despite the lowered metabolic costs at lower temperatures, much higher levels of clam SFG were recorded at higher SPM concentrations (> 45 mg L^–1^). Likewise, other bivalves such as *Ostrea edulis* and *Geukensia demissa* are able to compensate for the increased metabolic costs of warm temperatures by increasing their filtration rate [10,11]. In this respect, the present study demonstrates that high SPM concentration (i.e., high food availability) enables these animals to cope with the stress induced by temperature variations in the intertidal area [66]. The positive SFG of the clams across a broad range of environmental conditions indicates that they have a physiological plasticity that enables them to meet their physiological requirements for energy under highly variable environmental conditions, ensuring their somatic growth and further reproductive activity in intertidal habitats [17,34,67].

As a commercially important species, the Manila clam is now widely distributed throughout the world [68,69,70]. The clam is native to the subtropical to low boreal areas of the western Pacific and was introduced to Hawaiian waters in 1918. It was subsequently introduced into the Pacific coastal areas of North America in the 1930s, and into European and Mediterranean coastal areas since 1972, where it was first introduced off the coast of France. Subsequent successful colonization, reproduction, and production over such a wide variety of geographical areas attests to the ability of this species to adjust their physiological processes in a highly flexible way so that they are able to adapt to and thrive in a broad range of environmental conditions. Our findings support this hypothesis and show that physiological plasticity allows the clam to follow an adaptive strategy upon their introduction to new habitats off the North American, European, and Mediterranean coasts [68,69,70]. Furthermore, when the intertidal area is exposed to the air during neap tides in summer, the expected temperature elevation may cause a disruption in the physiological performance of clams. The increase in the aerobic metabolic component due to elevated temperatures during air exposure may alter energy budgets throughout whole tidal cycles in field conditions, leading to increased expenditure of energy reserves [71,72]. Therefore, although further research on the integrated effect of air exposure on the physiological energetics of the clams is needed [73], we expect that the results of our studies on native clam populations will help to clarify the mechanisms underlying the physiological adaptability of the introduced and subsequently naturalized populations in new environmental conditions such as those off the North American, European, and Mediterranean coasts.
